# Epidural Oxycodone for Acute Pain

**DOI:** 10.3390/ph15050643

**Published:** 2022-05-23

**Authors:** Panu Piirainen, Hannu Kokki, Merja Kokki

**Affiliations:** 1 Department of Anesthesiology, Surgery and Intensive Care, Oulu University Hospital, 90220 Oulu, Finland; panu.piirainen@ppshp.fi; 2 Institute of Clinical Medicine, School of Medicine, Faculty of Health Sciences, Kuopio Campus, University of Eastern Finland, 70210 Kuopio, Finland; hannu.j.kokki@gmail.com; 3 Department of Anaesthesiology and Intensive Care, Kuopio University Hospital, 70210 Kuopio, Finland

**Keywords:** epidural analgesia, oxycodone, pain, postoperative, pain, acute

## Abstract

Epidural analgesia is commonly used in labour analgesia and in postoperative pain after major surgery. It is highly effective in severe acute pain, has minimal effects on foetus and newborn, may reduce postoperative complications, and enhance patient satisfaction. In epidural analgesia, low concentrations of local anaesthetics are combined with opioids. Two opioids, morphine and sufentanil, have been approved for epidural use, but there is an interest in evaluating other opioids as well. Oxycodone is one of the most commonly used opioids in acute pain management. However, data on its use in epidural analgesia are sparse. In this narrative review, we describe the preclinical and clinical data on epidural oxycodone. Early data from the 1990s suggested that the epidural administration of oxycodone may not offer any meaningful benefits over intravenous administration, but more recent clinical data show that oxycodone has advantageous pharmacokinetics after epidural administration and that epidural administration is more efficacious than intravenous administration. Further studies are needed on the safety and efficacy of continuous epidural oxycodone administration and its use in epidural admixture.

## 1. Introduction

Severe acute pain is a common clinical problem during labour and after various surgical procedures [1]. Poorly controlled pain causes suffering and harmful physiological reactions, which may lead to a delay in mother–newborn bonding and in postoperative settings, to complications or even increased mortality [2,3,4].

In parturients, epidural analgesia is one of the most effective methods to relief contraction pain [5]. In labour analgesia, the benefit of epidural opioids relies on synergistic effects with local anaesthetics. By adding an opioid to a local anaesthetic, adequate labour analgesia is achieved with a lower local anaesthetic dose. Consequently, motor blockade and an inability to ambulate or push effectively during the second stage of labour are avoided [6].

In postoperative pain management, epidural analgesia is highly effective and commonly used in major abdominal and thoracic surgery. It offers excellent analgesia, blunts surgical stress response and may reduce postoperative complications, such as respiratory failure, ileus and delirium [7,8,9,10,11,12,13,14]. Epidural opioids are frequently co-administered with other compounds, most commonly with local anaesthetics, such as clonidine and adrenaline, to enhance analgesia, especially in the alleviation of dynamic pain [7,15].

The two opioids approved for epidural administration are morphine and sufentanil, but off-label use of neuraxial fentanyl is also common [15,16,17,18]. Oxycodone is one of the most commonly used opioids in postoperative analgesia and increasingly used in labour analgesia as well [19]. However, data on the epidural administration of oxycodone have been sparse until recent years [20].

The basic pharmacology of oxycodone has previously been reviewed [20,21]. The aim of this review is to evaluate the available literature on epidural administration of oxycodone. A systematic literature search was conducted in MEDLINE (PubMed) and Scopus in April 2022. The search strategy combined Medical Subject Heading (MeSH) terms and keywords supplemented with the text word-function. “Oxycodone” and “epidural” were used in combination to search for relevant studies. Nine clinical studies [22,23,24,25,26,27,28,29,30], two experimental studies in pregnant sheep [31,32] and three population pharmacokinetic (PK) studies [33,34,35] matched the inclusion criteria.

The data indicate that oxycodone is a feasible opioid in epidural analgesia. Compared to epidural morphine, pruritus and postoperative nausea and vomiting (PONV) may be reduced with epidural oxycodone. Further studies are needed on the safety and efficacy of repeated doses and continuous epidural infusion of oxycodone, on the use of oxycodone in epidural admixtures, and on direct comparisons with lipophilic opioids such as fentanyl and sufentanil.

## 2. Historical Aspects of the Pharmacology of Epidural Opioids

The clinical practice of spinal opioid administration for pain relief is based on observations in animals that opioid receptors exist not only in the supraspinal sites, but also in the substantia gelatinosa in the dorsal horn and in the dorsal root ganglia of the spinal cord [36,37]. Clinical use of epidural opioids began in the late 1970s, when analgesia with epidural morphine was introduced by Behar and colleagues [38].

Epidural morphine initially garnered interest because a single injection could produce long-lasting analgesia without hampering motor function, which is common with epidural local anaesthetics. However, late respiratory depression, assumedly caused by the rostral migration of morphine in cerebrospinal fluid (CSF), became a concern regarding epidural morphine soon after its introduction in clinical use. Thereafter, there has been interest in studying other opioids in epidural analgesia [39].

The lipophilic opioids fentanyl and sufentanil have become commonly used epidural opioids since the 1990s. Their epidural administration offers similar analgesia with a lower dose and less adverse drug events compared to intravenous (i.v.) administration [40,41,42,43]. Fentanyl and sufentanil have a relatively short duration of analgesic action after single injection, and that is why they are used mainly as continuous infusions [44,45].

Experimental studies in the 1990s and early 2000s have shown that liposolubility is a major determining factor in PK and the clinical effects of epidural opioids [46]. When opioids are injected epidurally, they must penetrate the meninges, CSF and spinal cord white matter to reach their site of action in the spinal cord. After epidural administration, opioids are also absorbed into epidural fat and into the systemic circulation. Lipophilic opioids accumulate into lipid-rich tissues such as epidural fat and spinal cord white matter. Consequently, their exposure in CSF and the extracellular space of the spinal cord is lower than that of hydrophilic opioids [46,47].

Studies in humans have found similar results. Lipophilic opioids are more readily cleared from CSF than hydrophilic opioids. The clearance from CSF is 27 µg min^–1^ kg^–1^ for sufentanil and 2.8 µg min^–1^ kg^–1^ for morphine after intrathecal administration [48,49].

This higher clearance from CSF has been assumed to explain why late respiratory depression is less common with lipophilic opioids. As lipophilic opioids are readily cleared from CSF, their cervical CSF concentrations are lower than those of morphine after epidural administration [50,51,52]. However, early respiratory depression is a concern with lipophilic opioids, which may be explained by rostral spread in CSF and absorption into the systemic circulation [52].

The CSF bioavailability of epidural opioids is substantially lower than that of local anaesthetics and α-2 adrenergic agonists (Table 1).

## 3. Oxycodone

### 3.1. General

Oxycodone is a commonly used opioid worldwide. Oxycodone has some advantages over morphine: higher per oral (p.o.) bioavailability, faster onset of analgesia, higher efficacy in visceral pain, less histamine release and fewer hallucinations [63,64,65,66]. Patient satisfaction is also higher in patients receiving oxycodone than in those receiving morphine [67]. Due to its high bioavailability, oxycodone is suitable for p.o. and transmucosal administration, and it is also given intramuscularly, i.v. and subcutaneously. In contrast to these administration routes, there have been limited data on the spinal use of oxycodone until recent years [20].

### 3.2. Experimental Animal Studies

The first data on neuraxial administration of oxycodone in animals were published in the early 1990s. Plummer (1990) and Pöyhiä & Kalso (1992) found that after intrathecal administration, the antinociceptive effect of morphine (hot-plate and tail-flick tests) was 14 times more potent than that of oxycodone in rats. The onset of the antinociceptive effect was faster with oxycodone but it was also of shorter duration compared to morphine. [68,69]. In line with earlier data in rats, it was later found that the median effective dose (ED50) for the antinociceptive effect (tail-flick test) for intrathecal oxycodone was 15 times higher than that of morphine in mice [70]. In contrast to intrathecal administration, after subcutaneous and intraperitoneal administration, oxycodone was 2–4 times more potent than morphine [69].

This discrepancy in the route-dependent antinociceptive efficacy of oxycodone is thought to result from its active uptake in the blood–brain-barrier (BBB) [71,72], but a relatively low µ-opioid receptor binding affinity and ability to activate the G-proteins have been shown in in vitro studies [70,73,74,75,76,77].

In rat central nervous system (CNS) tissue, oxycodone has a lower efficacy and potency to activate G-proteins than morphine and oxymorphone, especially in the periaqueductal grey and spinal cord. The antinociceptive efficacy and potency of intrathecal oxycodone is also lower compared to intrathecal oxymorphone [78].

Two recent experimental studies in 2018 and 2019 by Kinnunen and colleagues have evaluated the epidural administration of oxycodone. Pregnant sheep were given an epidural loading dose of 0.1 mg·kg^−1^ oxycodone followed by either a continuous infusion or repeated boluses of epidural oxycodone for five days. After five days, arterial blood samples were collected, the animals were killed and CSF and CNS tissue samples were obtained for analysis. Cervical CSF samples were obtained by cisternal magna punctures and CNS tissue samples were obtained from the cortex, thalamus, cerebellum and spinal cord. Oxycodone concentrations in the spinal cord were up to 400 times higher than brain concentrations. In humans, opioid concentrations in CSF are proposed as a surrogate of CNS exposure [79]. In the study by Kinnunen and colleagues, cervical CSF concentrations were similar to plasma oxycodone concentrations, and CSF concentrations correlated but did not predict tissue concentrations. Oxymorphone, one of oxycodone’s active metabolites, accumulated in the ewes’ CNS tissues and foetal plasma. These data suggest that epidural oxycodone can provide segmental spinal analgesic efficacy [31,32].

### 3.3. Clinical Studies

The first clinical study on epidural oxycodone was published in 1997 by Bäcklund and colleagues. In that study, 33 patients undergoing elective major abdominal surgery with combined epidural and general anaesthesia were randomised to receive either epidural morphine (*n* = 13) or epidural oxycodone (*n* = 16). In an open control group, 11 patients were given i.v. oxycodone at a similar dose (Table 2). Epidural morphine and oxycodone had similar analgesic efficacy at a dosing ratio of 1:8.4 to 1:9.8. Adverse drug events were similar between the epidural groups. Compared to epidural opioids, mild respiratory depression was more common in subjects receiving i.v. oxycodone. Postoperative pain scores were mainly similar between groups, but dynamic pain during coughing was more severe in subjects receiving epidural morphine than in the two oxycodone groups at 14 h postoperatively, and at 17 h, dynamic pain was more severe in subjects receiving i.v. oxycodone than in the two epidural groups [22].

There were limitations in the study by Bäcklund and colleagues. Firstly, the correct placement of an epidural catheter was not tested appropriately. Second, a prolonged, 3–5 h fentanyl infusion (2 µg kg^−1^ h^−1^) was used for intraoperative analgesia. This long intraoperative fentanyl infusion should have affected postoperative pain scores for several h, as the elimination half-life of fentanyl is relatively long (4 h). In addition, ketorolac was used for rescue analgesia, and the dose was substantially higher in the morphine group compared to the epidural oxycodone group. Lastly, the comparison between epidural morphine and epidural oxycodone was double-blinded whereas the i.v. oxycodone group was an open control, which renders the comparison between epidural and i.v. oxycodone inconclusive [22].

A later study by Yanagidate & Dohi (2004) found that epidural oxycodone and epidural morphine may be equianalgesic at a 1:2 ratio. In this study, 75 women undergoing elective gynaecological surgery with combined epidural and general anaesthesia were randomised to receive either epidural oxycodone or epidural morphine in a double-blinded manner (Table 2). Fentanyl boluses were used for intraoperative analgesia but the total doses were substantially lower (200 µg) than those in the study by Bäcklund and colleagues [22,29]. However, consistent with the study by Bäcklund and colleagues, the use of adjuvant analgesics was not standardised. In support of the feasibility of epidural oxycodone, adverse drug events, PONV and pruritus were less severe with epidural oxycodone than with epidural morphine [29].

A decade ago, Kokki and colleagues (2014) conducted a PK study on epidural and i.v. oxycodone in 24 women undergoing gynaecological surgery under general anaesthesia and epidural analgesia for postoperative pain management. At the end of surgery, the study subjects were randomised to receive a single bolus injection of oxycodone 0.1 mg kg^−1^ either epidurally or i.v. In both groups, a matching placebo injection was given by the other administration route to ensure blinding. Plasma and CSF samples were obtained via an indwelling cannula and a spinal catheter at multiple time points during the first 24 h to measure concentrations of oxycodone and its metabolites. The peak concentrations (maximum concentration, C_max)_ and area under the curve (AUC) in CSF after epidural administration were increased 320- and 120-fold compared to i.v. administration. The need for rescue analgesia was reduced in the epidural group compared to the i.v. group, supporting the superior analgesic efficacy of epidural oxycodone. [23]

In a small observational study, patients undergoing total hip arthroplasty (*n* = 11) had epidural anaesthesia with 15 mL bupivacaine 0.25% and oxycodone 5 mg. All patients were co-administered i.v. ketoprofen (a nonsteroidal anti-inflammatory drug, NSAID) 100 mg at every 12 h, and subcutaneous morphine for rescue analgesia when the pain score on an 11-point numerical rating scale (NRS) was >3. Analgesia lasted for a mean of 10 [minimum 5, maximum 24] h. One patient was given naloxone for pruritus and two patients had bradycardia, but otherwise epidural oxycodone was well tolerated. This small study with no control group renders it difficult to draw conclusions on whether oxycodone may have prolonged epidural bupivacaine analgesia or not, and whether the coadministration of oxycodone with bupivacaine is safe in this kind of patient population [24].

Sng and colleagues (2016) randomised *n* = 100 parturients undergoing caesarean section under spinal anaesthesia with hyperbaric bupivacaine 12 mg and fentanyl 15 µg to receive either epidural morphine 3 mg or epidural oxycodone 3 mg after delivery in a double-blind manner. Per oral paracetamol 1 g three times daily (tds), mefenamic acid (NSAID) 500 mg tds and tramadol 50 mg as needed were used for multimodal analgesia. The need for rescue tramadol during the first 24 postoperative h was higher (*n* = 9 (18%) vs. *n* = 2 (4%)) and pain scores were higher in the oxycodone group compared to the morphine group, but patient satisfaction was similarly high in the two groups. Two patients in the morphine group need treatment for pruritus and two patients had antiemetics for protracted PONV [27].

Zhong and colleagues (2020) randomised 40 parturients to receive 10 mL of epidural ropivacaine 1 mg mL^−1^ with or without oxycodone 2 mg for labour analgesia in a double-blind study. An epidural test dose of 3 mL lidocaine 15 mg mL^−1^ was used to exclude inadvertent intrathecal catheterization, but as no adrenaline was used, the test dose did not exclude inadvertent i.v. catheterization. Appropriate location of the catheter in epidural space was determined by loss of sensation to cold at Th10 dermatome and achievement of a visual analogue pain (VAS) score < 3 (onset of analgesia). Pain scores were similar between the two groups for the first 2 h, but thereafter until the time of cervical dilatation of 10 cm, pain scores were lower in the oxycodone group. The oxycodone group also needed fewer epidural boluses during the study and the time to the first dose of rescue analgesia (VAS > 3) was 5–6 h longer in the oxycodone group than in the control group, 6.5 h and 1.1 h, respectively. Four parturients in the oxycodone group but none in the control group had pruritus. Otherwise, there were no differences in adverse drug events [30].

Recent randomised clinical trials (RCTs) have indicated that the analgesic efficacy of epidural oxycodone is superior to i.v. oxycodone in postoperative analgesia [25,26]. Ninety women undergoing gynaecological surgery under general anaesthesia were randomised to epidural or i.v. oxycodone 0.1 mg kg^−1^ postoperatively. Background multimodal analgesia was standardised; i.v. paracetamol 1 g tds and i.v. dexketoprofen 50 mg tds; and rescue analgesia i.v. fentanyl 50 µg. After the first 4 h, epidural infusion of levobupivacaine, fentanyl and adrenaline were initiated for all patients. The primary outcome measure was the need for rescue analgesia during the first 4 postoperative h. Pain scores were measured at rest as well as dynamic pain during coughing and during wound compression. The need for rescue i.v. fentanyl during the first 4 postoperative h was lower after epidural oxycodone than after i.v. oxycodone, regardless of surgical technique, laparotomy (*n* = 30) [25] or laparoscopy (*n* = 60) [26]. Resting and dynamic pain scores were lower in the epidural group during the first postoperative h, but similar between groups thereafter. Adverse drug events were similar in the two groups. PONV was most common, *n* = 18 and *n* = 18 in the two groups respectively. Eight women in the epidural groups and 6 women in the i.v. groups had a respiratory rate < 10 min^−1^, but none of them needed any interventions. Pruritus was reported to be two times more common in the epidural group, with 52%, compared to the i.v. group with 23%.

In these two double-blind, double-dummy, randomised studies the correct placements of epidural catheters were verified by administering lidocaine 50 mg with adrenaline 50 µg, and testing the loss of sensation to cold. Moreover, intraoperative analgesia was provided with remifentanil infusion, and the infusion rate was adjusted with the Surgical Pleth Index (Carescape™ B650; GE Healthcare, Helsinki, Finland). As the elimination half-life of remifentanil is 12–30 min, it is unlikely that the intraoperative opioid had affected recovery after surgery very much [80]. As the background multimodal analgesia was also strictly standardised, the authors concluded that their results should be on a sound basis [25,26].

A recent double-blind, three-arm dose-response study assessed whether adding oxycodone to epidural ropivacaine may decrease the dose of local anaesthetics required for surgical anaesthesia. Patients (*n* = 141) undergoing high ligation and stripping of the great saphenous vein were allocated to two active groups: epidural oxycodone 2.5 mg and epidural oxycodone 5.0 mg, and to a control group with no additive to ropivacaine. The volume of epidural injection was 15 mL in all three groups. The median effective concentration (EC50) of ropivacaine was assessed by an up-and-down sequential method. In the two oxycodone groups, the EC50 of ropivacaine was 4 mg/mL, significantly less than that in the placebo group with 5 mg/mL. A clinically meaningful finding was a relatively short duration of analgesic action in the epidural oxycodone 5 mg group; time to VAS score >4 was 3 h after epidural injection compared to 9 h in the control group and 10 h in the epidural oxycodone 2.5 mg group [28].

### 3.4. Population Pharmacokinetics

Population pharmacokinetic–pharmacodynamic (PK-PD) modelling improves our understanding of analgesic drug action, explains interindividual variability and improves our ability to titrate drugs to the desired effect [81]. A few population PK models have been published for epidural oxycodone.

A five-compartment model described the time concentration data of i.v. and epidural oxycodone accurately and precisely. The epidural space served as a depot compartment, from which the drug could transfer into the central compartment and CSF compartment. Covariates did not correlate with PK parameters and were not modelled [33]. Another population PK model with three compartments suggested that 60% of epidural oxycodone initially penetrates into CSF and 40% is absorbed into the systemic circulation [26].

A universal population PK model was recently developed to describe oxycodone plasma concentrations across a wide range of ages and body sizes after i.v., intramuscular, buccal, nasogastric and epidural administration. A three-compartment model with first-order elimination best described the data. Clearance matured with age, reaching 90% of typical adult values by the age of one year. Allometric scaling using total body weight better explained clearance than fat-free mass [35]. This model enabled the authors to give oxycodone dose recommendations to achieve analgesic concentrations in plasma [34]. Previously, two- or three-compartment models have been used to describe oxycodone time concentration data after i.v. administration [82,83].

## 4. Glymphatic Pathway and Epidural Analgesia

**Figure 1:**
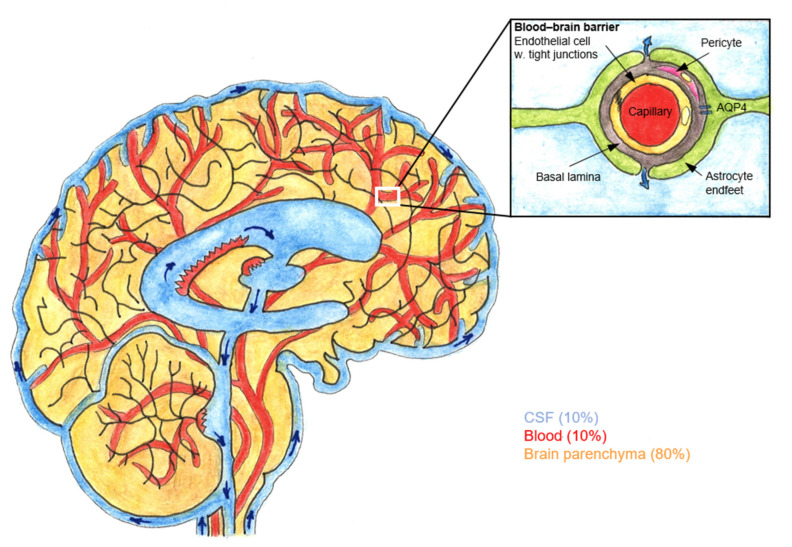
The blood–brain barrier (BBB) and glymphatic pathway. Sagittal section of brain and the surrounding subarachnoid space containing cerebrospinal fluid (CSF). The location of the perivascular space is between the endothelial cells and astrocytic endfeet. CSF flows from the subarachnoid space into the arterial perivascular spaces. From the perivascular space, CSF diffuses into brain parenchyma via astrocytic endfeet that express water channel aquaporin 4 (AQP4). Adapted from Jessen et al. (2015). Drawn by Lassi Piirainen.

A recently discovered fluid transport system, the glymphatic pathway, may enhance our understanding of the CNS bioavailability of intrathecal drugs and have implications for epidural analgesia (Figure 1).

The glymphatic pathway enables solute clearance from the brain’s extracellular space. and impaired function of the glymphatic system may contribute to the pathogenesis of several chronic neurodegenerative diseases and to poor recovery after acute brain insults [84,85].

Some physiological factors, such as sleep and aerobic exercise, enhance glymphatic pathway activity, which seems to be linked to an expansion of the brain’s interstitial fluid space as well as slow-wave delta oscillations [86,87,88]. Experimental studies in rodents have shown that some general anaesthetics, such as dexmedetomidine and ketamine/xylazine (α-2 adrenergic agonist), and osmotic interventions, such as hypertonic saline, enhance glymphatic transport [89,90].

The glymphatic pathway is active not only in the brain, but also in the spinal cord [91]. Drugs that enhance glymphatic flow could be used to facilitate better CNS distribution of intrathecal drugs. In experimental studies in rats, subcutaneous and intrathecal administration of dexmedetomidine increased the brain and spinal cord concentrations of intrathecal oxycodone and naloxone, but not those of morphine [92]. More recently, an experimental study on rodents showed that hypertonic saline enhances the spinal cord penetration and antinociception of intrathecal morphine [93]. These experimental findings are yet to be confirmed in humans. However, it is well established that i.v. dexmedetomidine prolongs spinal and epidural anaesthesia and analgesia, which could be explained by an enhancement of glymphatic flow and spinal cord penetration of local anaesthetics [94,95]. Whether the spinal cord penetration of epidural oxycodone, other opioids or local anaesthetics could also be improved by enhancement of glymphatic flow remains to be elucidated.

## 5. Discussion

If a new drug is to be introduced to epidural analgesia, it is a prerequisite that the drug is safe for spinal administration and secondly, more efficacious when given epidurally than i.v. [96]. Recent clinical studies have shown that epidural oxycodone is substantially more efficacious than the same dose given i.v. [23,25,26]. Epidural oxycodone seems to be equipotent to epidural morphine at a dosing ratio of 2–10:1 [22,29].

The onset of analgesia with epidural oxycodone is faster than with epidural morphine. After a bolus dose of epidural oxycodone, the onset of analgesia is about 30 min, which is consistent with the median time to maximum concentration in CSF (t_max-CSF_) of 36 min [25,26] (Table 3). This is faster than what has been reported for morphine, with the onset of analgesia occurring at 30 to 90 min after epidural morphine injection and t_max-CSF_ between 60 and 90 min [58,97,98]. However, for epidural morphine t_max-CSF_ as long as 3.6 h has been reported [99]. Compared to the lipophilic epidural opioids sufentanil and fentanyl, the onset of analgesia is slower; it is 5 to 10 min with epidural sufentanil and 10 to 15 min with epidural fentanyl [44,45]. The onset of analgesia with epidural sufentanil is considerably faster than the t_max-CSF_ of 0.8–2.1 h, which suggests that the rapid onset of analgesic action of epidural sufentanil may be partly explained by rapid systemic absorption and supraspinal effects [59] (Table 3).

The duration of analgesic action is about 3 h with a single bolus dose of epidural oxycodone for postoperative pain after abdominal surgery [25,26]. This is similar to epidural sufentanil and fentanyl but substantially less than with a single bolus of epidural morphine, which provides analgesia up to 12–24 h [45]. The relatively short duration of action with epidural oxycodone is unexpected, because the liposolubility of oxycodone is similar to that of morphine, with an octanol-water partition coefficient of 0.7 for oxycodone and 0.5 for morphine [100]. Moreover, the dose-normalized AUC_CSF_ of epidural morphine and epidural oxycodone are similar after a single bolus [23,99] (Table 3). This discrepancy between favourable PK characteristics but a relatively short duration of action of epidural oxycodone in comparison to epidural morphine may be explained by PD differences of these two drugs. In experimental studies in rats, onset and duration of analgesia were shorter with oxycodone than with morphine. Oxycodone was also less potent after intrathecal administration in rats and had a lower efficacy and potency in activating G-proteins in rat spinal cord tissue samples [69,78].

Co-administration with local anaesthetics may prolong the analgesic action of epidural oxycodone. In labour analgesia, analgesia lasts for 1.2 to 1.5 h after i.v. oxycodone administration, but in epidural analgesia, when oxycodone 3 mg is co-administered with ropivacaine 15 mg, the duration of analgesia is 4 times longer [30]. In a study by Zhong and colleagues (2020), analgesia achieved with epidural ropivacaine without oxycodone was also short, 1.2 h, which supports the data with other opioids suggesting that analgesia is more effective when epidural opioids are used in combination with local anaesthetics [19,30,101]. In clinical practice, it would be reasonable to co-administer oxycodone as a continuous infusion or as repeated bolus doses with epidural local anaesthetics and adrenaline or α-2 adrenergic agonists. The established population PK models for epidural oxycodone may help to optimize dosing regimens in further clinical trials and clinical practice [26,33,35].

Both clinical and experimental data are needed to establish the safety and efficacy of spinal oxycodone administered as a single compound and in admixtures. Epidural opioids and α-2 adrenergic agonists exert their analgesic effect by binding to receptors in the spinal cord dorsal horn. Ideally, drug concentrations would be measured at their site of action, but such data can be obtained only in experimental animal studies [47]. After epidural administration, opioids and α-2 adrenergic agonists are assumed to diffuse firstly into CSF and then into the superficial dorsal horn of the cord [102]. Thus, CSF drug concentrations may be used to predict analgesic effects and epidural drug doses that produce the desired effect. For example, computer-controlled epidural infusion of clonidine has been shown to maintain steady drug concentrations in CSF [103].

The next step in PK-PD modelling of epidural oxycodone would be to assess the EC50 value in CSF. This could be achieved by PK-PD-modelling the relationship between exposure of epidural oxycodone and rescue fentanyl consumption using repeated time-to-event (RTTE) modelling in patients undergoing gynaecological surgery [25,26,33,104]. Thereafter, the published population PK models could be utilized to determine the dose of epidural oxycodone which produces sufficient (above EC50) CSF concentrations.

PK modelling may also improve safety with epidural oxycodone. A recently published population PK model enabled the authors to give dosing recommendations for epidural oxycodone that produce analgesic plasma concentrations without an increased risk of adverse effects from respiratory depression [34]. Oxycodone is metabolized mainly in the liver by CYP3A4 and CYP2D6. Drug–drug interactions between oxycodone and CYP inducers/inhibitors may be simulated with PK modelling, as has been done with epidural fentanyl [105].

The high C_max-CSF_ and AUC_CSF_ of oxycodone raise the concern of late respiratory depression, as shown with epidural morphine. However, to our knowledge, late respiratory depression has not been reported with epidural oxycodone. Mild early respiratory depression was observed in a few patients after epidural oxycodone, but the frequency was similar to that of i.v. oxycodone. Moreover, in the studies by Piirainen and colleagues, all patients with a low respiratory rate < 10·minute^−1^ had also received i.v. fentanyl for rescue analgesia [25,26]. In experimental studies in sheep receiving continuous infusion or repeated boluses of epidural oxycodone over multiple days, the spinal cord concentrations of oxycodone at the epidural catheter tip level were up to 400 times greater than brain and cervical CSF concentrations [32]. This may imply that rostral spread in CSF is limited with epidural oxycodone. However, more safety data on epidural data are needed, and close follow-up is always required when opioid analgesics are administered by any administration route.

Consistent with other spinal opioids, pruritus is more common with epidural oxycodone than with i.v. oxycodone. However, both pruritus and PONV seems to be reduced with epidural oxycodone compared to epidural morphine [27,29]. Pruritus and PONV are less common with epidural fentanyl than with epidural morphine, but there are no studies comparing epidural oxycodone to epidural fentanyl or sufentanil [106].

Neurotoxicity is a concern with any epidural drug and every opioid evaluated so far has shown at least some degree of neurotoxicity. However, very little neurotoxicity is reported in daily clinical practice with epidurally administered opioid doses when conventional doses are utilized [96]. The neurotoxicity of oxycodone has been studied in one in vitro study. In human neuroblastoma cells and mouse motoneuronal cells, the neurotoxicity of oxycodone was similar or less than that of morphine [107]. More safety data for all epidural opioids are needed for prolonged infusions as are used in chronic pain conditions. Severe neurologic damage has been reported with epidural morphine and hydromorphone in a sheep model with 30-day infusion of high opioid concentrations [108]. Postmortem examination of spinal cords from patients who had received prolonged infusions of intrathecal morphine revealed no damage, indicating that spinal cord damage following the administration of preservative free epidural opioids is extremely rare [109].

## 6. Conclusions

Oxycodone is a feasible opioid for epidural analgesia. Pruritus and PONV may be reduced with epidural oxycodone compared to epidural morphine. The established population PK models may facilitate effective and safe dosing recommendations for clinical practice.

## 7. Future Directions

Further studies should assess the safety and analgesic efficacy of epidural oxycodone with local anaesthetics and adrenaline or α-2 adrenergic agonists. In addition, head-to-head comparisons with epidural lipophilic opioids are required. The neurotoxicity of epidural oxycodone should be studied in animal trials before facilitating the clinical use of epidural oxycodone. A better understanding of the interplay between the glymphatic pathway and intrathecal drugs may open new possibilities to treating different pain states more safely and effectively.

## Figures and Tables

**Table 1:** Cerebrospinal fluid (CSF) bioavailability of local anaesthetics, opioids and α-2 adrenergic agonists after epidural administration. F_CSF_, CSF bioavailability; t_max-csf_, time to maximum concentration in CSF.

Drug	Species	F_csf_ (%)	t_max-csf_ (min)	Reference
Lidocaine	Rabbit	18	7.0	[53]
Bupivacaine	Rabbit	13	6.8	[54]
	Rabbit	5.5	5.6	[53]
Ropivacaine	Rabbit	11	6.8	[54]
	Sheep	11	12	[55]
Morphine	Goat	2.3–11 *	13	[56]
	Man	3.2 1.9 3.6	56 135 80	[49,57,58]
Sufentanil	Man -bolus -infusion	2.7 0.4–0.7	46–126 **	[59,60]
Fentanyl	Goat	0.8–3.3 *	13	[56]
Clonidine	Sheep	14	32	[61]
Dexmedetomidine	Sheep	22	12	[62]

* CSF bioavailability was measured after two doses (low and high) of epidural morphine (4 and 8 mg) and epidural fentanyl (0.1 and 0.2 mg); ** t_max-csf_ in lumbar CSF was 46 min after lumbar epidural sufentanil bolus and 126 min after thoracic epidural sufentanil bolus.

**Table 2:** Medication protocols in randomised controlled trials (RCT) on analgesic efficacy of epidural (epid.) oxycodone. i.v., intravenous.

Study	Epidural Group 1	Epidural Group 2	Control Group
Bäcklund et al., 1997	Epid. oxycodone 0.15 mg kg^−1^ + infusion 0.03 mg kg^−1^ h^−1^	Epid. morphine 0.015 mg kg^−1^ + infusion 0.003 mg kg^−1^ h^−1^	i.v. oxycodone 0.15 mg kg^−1^ + infusion 0.03 mg kg^−1^ h^−1^
Yanagidate and Dohi 2004	Epid. oxycodone 2 mg with 25 mg bupivacaine + oxycodone infusion 6 mg d^−1^	Epid. oxycodone 4 mg with 25 mg bupivacaine 10 mL + oxycodone infusion 12 mg d^−1^	Epid. morphine 2 mg with 25 mg bupivacaine 10 mL + morphine infusion 6 mg d^−1^
Piirainen et al., 2018	Epid. oxycodone 0.1 mg kg^−1^		i.v. oxycodone 0.1 mg kg^−1^
Piirainen et al., 2019	Epid. oxycodone 0.1 mg kg^−1^		i.v. oxycodone 0.1 mg kg^−1^
Xie et al., 2022	Epid. oxycodone 2.5 mg with ropivacaine in 15 mL	Epidural oxycodone 5 mg with ropivacaine in 15 mL	Epidural ropivacaine 15 mL

**Table 3:** Plasma and cerebrospinal fluid (CSF) and pharmacokinetics (PK) of epidural oxycodone and the commonly used hydrophilic opioid morphine and the lipophilic opioid sufentanil after lumbar and thoracic epidural administration. Oxycodone 0.1 mg kg^−1^, morphine 2 mg and sufentanil 75 µg were diluted in 10 mL saline. Data are median (range) for oxycodone and mean (standard deviation) for morphine and sufentanil. Adapted from Kokki et al. (2014), Nordberg et al. (1987) and Hansdottir et al. (1995). AUC, area under the curve; AUC_CSF_ area under the curve in CSF; C_max_, maximum concentration; C_max-CSF_, maximum concentration in CSF; t_max_, time to maximum concentration; t_max-CSF_, time to maximum concentration in CSF; t_½_, elimination half-life; t_½-CSF_, elimination half-life in CSF.

Variable	Morphine		Sufentanil		Oxycodone
	Administration Site		Administration Site		Administration Site
	L2–3	Th7–8	L2–3 or L3–4	Th5–6 or Th6–7	Th12–L1 or L1–2
Plasma	mean (SD)		mean (SD)		median (range)
C_max_ (ng·mL^−1^)	16.3 (2.5)		0.40 (0.14)	0.26 (0.15)	29 (14–77)
t_max_ (h)	0.15 (0.08)		0.12 (0.12)	0.27 (0.20)	2.1 (0.6–4.2)
AUC (ng·h·mL^−1^)			1.2 (0.4)	1.5 (0.3)	201 (140–500)
t_½_ (h)			4.1 (1.2)	6.3 (2.9)	3.8 (3.1–5.1)
CSF lumbar	L3–4		L3–4 or L4–5		L3–4
C_max-CSF_ (ng·mL^−1^)	390 (139)	206 (120)	17.8 (29.6)	2.2 (4.9)	10,000 (982–10,000) *
C_max-CSF_ dose^−1^ (ng·mL^−1^)	2.0 × 10^−4^ (7.0 × 10^−5^)	1.0 × 10^−4^ (6 × 10^−5^)	2.4 × 10^−4^ (4.0 × 10^−4^)	2.9 × 10^−5^ (6.5 × 10^−5^)	1.6 × 10^−3^ (1.5 × 10^−4^–1.6 × 10^−3^)
t_max-CSF_ (h)	2.4 (2.9)	3.6 (2.3)	0.8 (0.5)	2.1 (1.4)	0.6 (0.2–4.0)
AUC_CSF_ (ng·h·mL^−1^)	2700 (925)	1370 (465)	22.9 (25.8)	4.9 (7.9)	23,000 (8300–42,000)
AUC_CSF_ dose^−1^ (ng·h·mL^−1^)	1.4 × 10^–3^ (4.6 × 10^−4^)	6.9 × 10^–4^ (2.3 × 10^−4^)	3.1 × 10^–4^ (3.4 × 10^−4^)	6.5 × 10^–5^ (1.1 × 10^−4^)	3.6 × 10^−3^ (1.3 × 10^−3^–6.6 × 10^−3^)
t_½-CSF_ (h)			2.8 (0.9)		

* Oxycodone concentrations were above the limit of quantification (10,000 ng mL^−1^) in 24 CSF samples in 7 subjects receiving epidural oxycodone.

## Data Availability

Data sharing not applicable.
